# Cardiac herniation after extrapleural pneumonectomy

**DOI:** 10.1093/jscr/rjaa011

**Published:** 2020-02-21

**Authors:** Natsumasa Nishizawa, Toshihiro Osaki, Yukiko Fukuichi, Manabu Yasuda

**Affiliations:** Department of Chest Surgery, Iizuka Hospital, Iizuka, Fukuoka, Japan

## Abstract

Cardiac herniation is a fatal complication in patients undergoing pneumonectomy with pericardial resection. A 53-year-old man underwent right-sided extrapleural pneumonectomy for malignant pleural mesothelioma. He underwent right-sided pericardial resection and reconstruction with an expanded polytetrafluoroethylene sheet. Routine chest radiography performed 18 h postoperatively revealed cardiac herniation into the right-sided thoracic cavity. The patient was immediately transferred to the operating room, and the defect was repaired. He died of tumor progression. However, cardiac herniation did not recur over 2 years postoperatively.

## INTRODUCTION

Extrapleural pneumonectomy is a surgical technique used to treat malignant pleural mesothelioma. However, it is associated with several complications, such as massive bleeding, heart failure, dyspnea and electrocardiographic abnormalities. Cardiac herniation is a rare and fatal complication because it precipitates acute cardiac failure and injury to the heart or vessels. We present a case of postoperative right cardiac herniation after an extrapleural pneumonectomy.

## CASE REPORT

A 53-year-old man presented to our hospital with cough and a mediastinal tumor, and based on pleural biopsy, he was diagnosed with a malignant pleural mesothelioma, clinical stage III (T2 N1 M0). He received two courses of chemotherapy with cisplatin and pemetrexed, after which we performed right-sided extrapleural pneumonectomy with pericardial resection, and pericardial and diaphragmatic reconstruction with expanded polytetrafluoroethylene (ePTFE) sheets using interrupted non-absorbable monofilament sutures.

**Figure 1:**
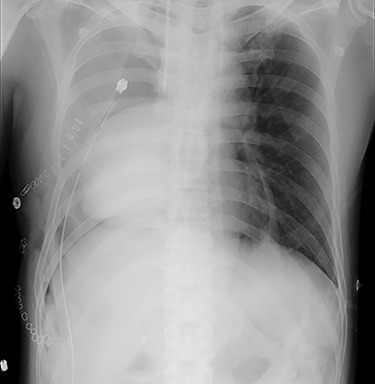
Chest radiography showing cardiac herniation into the right-sided thoracic cavity.

**Figure 2:**
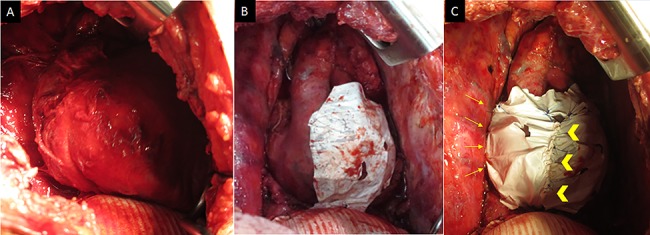
(**A**) Intraoperative images (at the time of the second operation) showing the patient’s heart is completely herniated into the thoracic cavity. (**B**) The heart is restored into the pericardium, and complete tearing of sutures is observed on the posterior aspect. (**C**) Pericardial repair performed by fixing another ePTFE sheet to the defect between the posterior pericardium and the preciously placed ePTFE sheet using horizontal mattress sutures on the posterior aspect (arrows) and a continuous suture between both sheets (arrow head).

He was transferred to the postoperative care unit and no significant symptoms and hemodynamic impairment were observed at that time. He complained of feeling unwell 18 h postoperatively and developed hypotension (89/57 mmHg with noradrenaline 0.015 μg/kg/min). Routine chest radiography revealed unexpected cardiac herniation into the right-sided thoracic cavity (Fig. 1). The patient was immediately transferred to the operating room and underwent emergency thoracotomy. Cardiac herniation had occurred secondary to tearing of sutures on the posterior aspect of the ePTFE sheet. We placed the heart back within the pericardium and repaired the tears by fixing another ePTFE sheet to close the defect between the posterior pericardium and the previously placed ePTFE sheet using horizontal mattress and continuous sutures (Fig. 2). The postoperative course was uneventful, and he was discharged 43 days after second operation. He died of tumor progression 2 years after the second operation. However, Cardiac herniation did not recur postoperatively.

## DISCUSSION

Since first reported by Bettman [1], cardiac herniation is recognized as a fatal complication of intrapericardial or extrapleural

pneumonectomy. Clinically, patients present with cardiac arrest, bleeding, hypotenshion, cyanosis, tachycardia, varicosis or superior vena cava syndrome and/or various electrocardiographic abnormalities [2, 3]. Fukui [2] reported 76 patients with cardiac herniation of which 71 patients (93.4%) developed cardiac herniation within 24 h. Fifty-four (71.1%) showed right-sided herniation, 69 (90.8%) underwent re-thoracotomy and pericardiotomy (11 cases), direct suture (8 cases), patch or mesh closure (49 cases) and others (‘repair the fat flap’ and ‘none declared’) and 14 (18.4%) died from cardiac shock by herniation or heart failure and infection secondary to herniation. Deiraniya [3] also reported 25 patients of which all patients developed cardiac herniation within 24 h (from ‘the time at conclusion of operation’ to ‘return to recovery room’ in the most cases), 14 (56.0%) showed right-sided herniation, 21 (84.0%) underwent re-thoracotomy and 12 (48.0%) died from cardiac herniation (one patient died from gastric bleeding). An abnormal position of the cardiac shadow observed on chest radiography indicates cardiac herniation in most cases. Rodgers [4] reported usefulness of thoracoscopy to diagnose left-sided cardiac herniation following radical pneumonectomy. Although, Bettman [1] reported wide pericardial excision in such cases, Sharma [5] showed that this procedure was unsatisfactory owing to recurrence observed in patients who underwent such resection. Recently, the pericardial defect is usually reconstructed with direct suturing or synthetic patches, such as ePTFE sheets [2]. However, Misawa [6] reported ePTFE sheet induced constrictive pericarditis in a few cases. Reconstruction with direct suturing should be considered in cases of small cardiac defects.

The present case developed hypotension, and fortunately, we could diagnose this condition based on routine postoperative chest radiographs. The high tension of the ePTFE sheet and interrupted sutures used on the posterior aspects of the sheet during the initial operation resulted in tearing of the posterior sutures. We used another ePTFE sheet and horizontal mattress sutures to release the tension on the posterior pleura and the ePTFE sheets to avoid re-tearing. We concluded that the thoracic cavity was large, and thoracic tissues were fragile. Therefore, careful and close observation is warranted to detect acute cardiac herniation, and it is necessary to use a large patch with light release of tension.

In conclusion, cardiac herniation occurring after extrapleural pneumonectomy is often fatal. However, reconstruction of the pericardial defect along with light release of tension at the initial operation can prevent this complication.
